# Biphenyls and dibenzofurans of the rosaceous subtribe Malinae and their role as phytoalexins

**DOI:** 10.1007/s00425-023-04228-7

**Published:** 2023-09-09

**Authors:** Belnaser A. Busnena, Ludger Beerhues, Benye Liu

**Affiliations:** Institute of Pharmaceutical Biology, Technische Universität Braunschweig, Mendelssohnstraße 1, 38106 Braunschweig, Germany

**Keywords:** Antimicrobial activity, Apple replant disease, Biosynthesis, Inducers, Structural diversity, Rosaceae

## Abstract

**Main conclusion:**

Biphenyl and dibenzofuran phytoalexins are differentially distributed among species of the rosaceous subtribe Malinae, which includes apple and pear, and exhibit varying inhibitory activity against phytopathogenic microorganisms.

**Abstract:**

Biphenyls and dibenzofurans are specialized metabolites, which are formed in species of the rosaceous subtribe Malinae upon elicitation by biotic and abiotic inducers. The subtribe Malinae (previously Pyrinae) comprises approximately 1000 species, which include economically important fruit trees such as apple and pear. The present review summarizes the current status of knowledge of biphenyls and dibenzofurans in the Malinae, mainly focusing on their role as phytoalexins. To date, 46 biphenyls and 41 dibenzofurans have been detected in 44 Malinae species. Structurally, 54 simple molecules, 23 glycosidic compounds and 10 miscellaneous structures were identified. Functionally, 21 biphenyls and 21 dibenzofurans were demonstrated to be phytoalexins. Furthermore, their distribution in species of the Malinae, inhibitory activities against phytopathogens, and structure–activity relationships were studied. The most widely distributed phytoalexins of the Malinae are the three biphenyls aucuparin (**3**), 2ʹ-methoxyaucuparin (**7**), and 4ʹ-methoxyaucuparin (**9**) and the three dibenzofurans *α*-cotonefuran (**47**), *γ*-cotonefuran (**49**), and eriobofuran (**53**). The formation of biphenyl and dibenzofuran phytoalexins appears to be an essential defense weapon of the Malinae against various stresses. Manipulating phytoalexin formation may enhance the disease resistance in economically important fruit trees. However, this approach requires an extensive understanding of how the compounds are formed. Although the biosynthesis of biphenyls was partially elucidated, formation of dibenzofurans remains largely unclear. Thus, further efforts have to be made to gain deeper insight into the distribution, function, and metabolism of biphenyls and dibenzofurans in the Malinae.

## Introduction

**Fig. 1:**
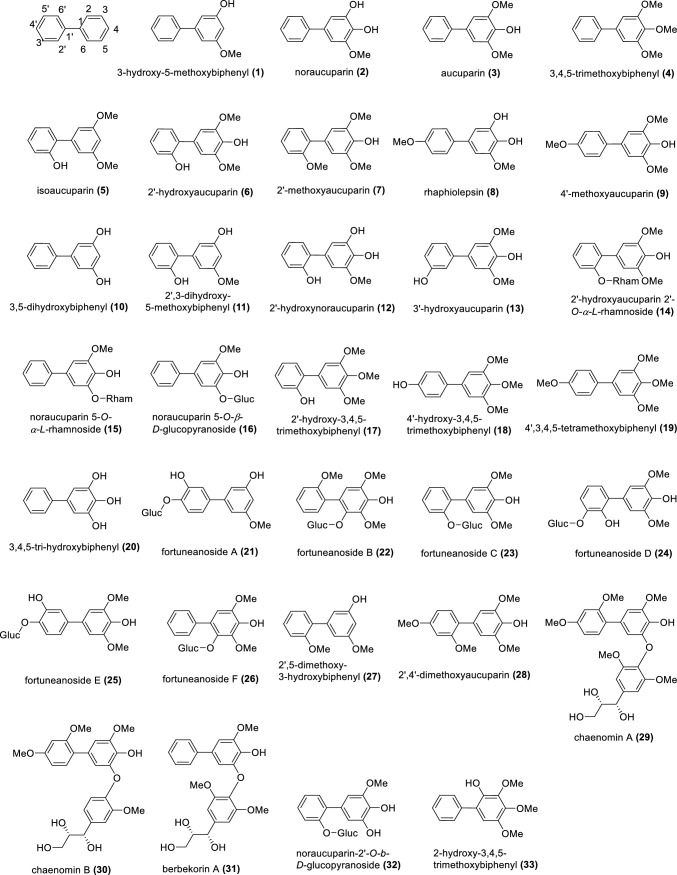

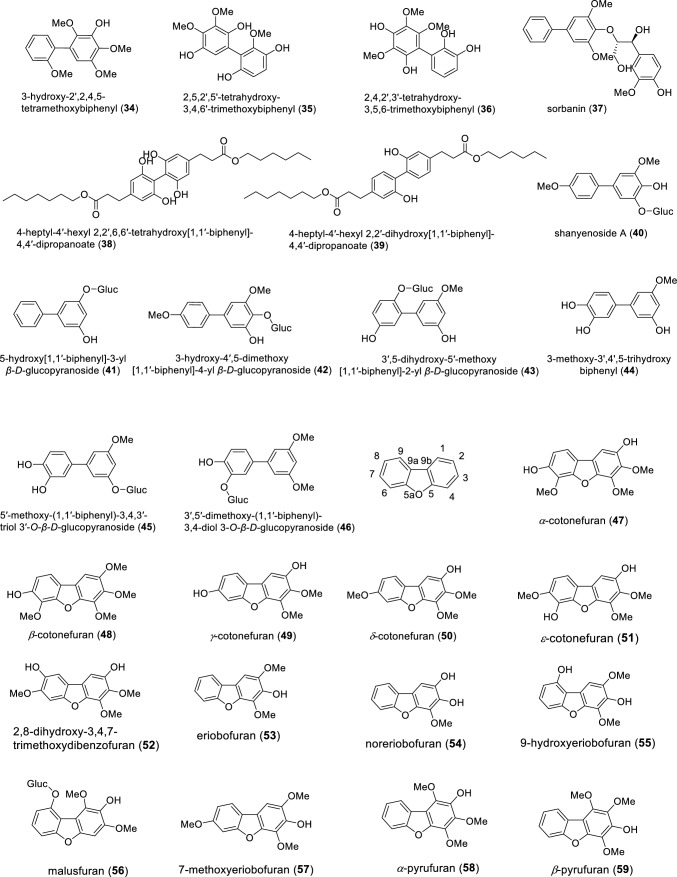

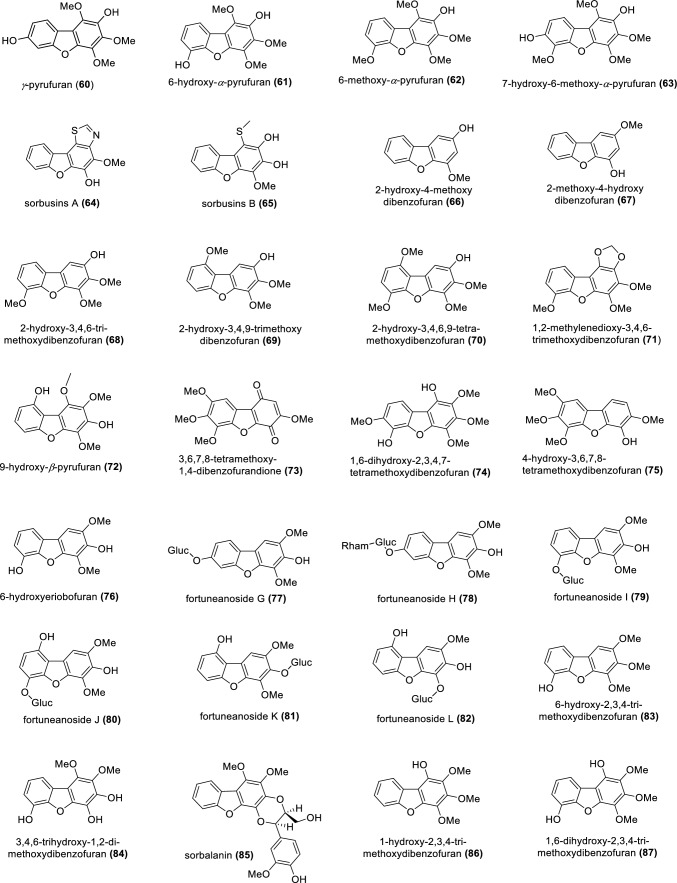
Biphenyls (**1–46**) and dibenzofurans (**47–87**) isolated from species of the rosaceous subtribe Malinae

The subtribe Malinae (previously Pyrinae) of the subfamily Amygdaloideae within the Rosaceae consists of about 30 genera and approximately 1000 species and includes economically important fruit trees, such as apple (*Malus domestica*), pear (*Pyrus communis*), and quince (*Cydonia oblonga*) (Sun et al. 2018; Ulaszewski et al. 2021). The major phytoalexins of the Malinae are biphenyls and dibenzofurans (Watanabe et al. 1982; Kemp et al. 1983; Kokubun and Harborne 1995). Phytoalexins are de novo formed specialized metabolites exhibiting antimicrobial activity. They accumulate in plants after exposure to biotic and abiotic stresses as a component of a complex system of disease resistance (Jeandet 2015; Bizuneh 2021). Biphenyls and dibenzofurans are biogenically related phenolic compounds. Biphenyls consist of two benzene rings connected by a single C–C bond, whereas dibenzofurans have two benzene rings connected by a furan ring (Fig. 1). In the Malinae, the core structures of both classes of compounds are usually decorated by hydroxy, methoxy, sugar (glucose and/or rhamnose), and other moieties. The last review of biphenyl and dibenzofuran phytoalexins of the Malinae was published over ten years ago. It reported 10 biphenyl and 17 dibenzofuran phytoalexins, which were isolated from 29 species belonging to 14 Malinae genera (Chizzali and Beerhues 2012).

Here, a survey was conducted by going through large electronic databases, such as PubMed, Google Scholar, and SciFinder etc. Based on these literature searches, 46 biphenyls and 41 dibenzofurans were detected in 44 plant species, which belong to 14 genera of the subtribe Malinae. Of the recorded compounds, 21 biphenyls and 21 dibenzofurans were formed in response to biotic and/or abiotic inducers. Thus, they were considered as phytoalexins. We summarize the chemical diversity of the reported biphenyls and dibenzofurans and their distribution among the Malinae species. Progress in elucidating their biosynthetic pathway is also presented. In the context of phytoalexin induction conditions, apple replant disease (ARD) is introduced as an in vivo model for eliciting the formation of biphenyl and dibenzofuran phytoalexins in apple roots. Finally, new information is added to the current knowledge of the antimicrobial activity of the phytoalexins, particularly regarding their inhibitory effect on phytopathogens.

## Chemical diversity and distribution of biphenyls and dibenzofurans in the subtribe Malinae

**Fig. 2:**
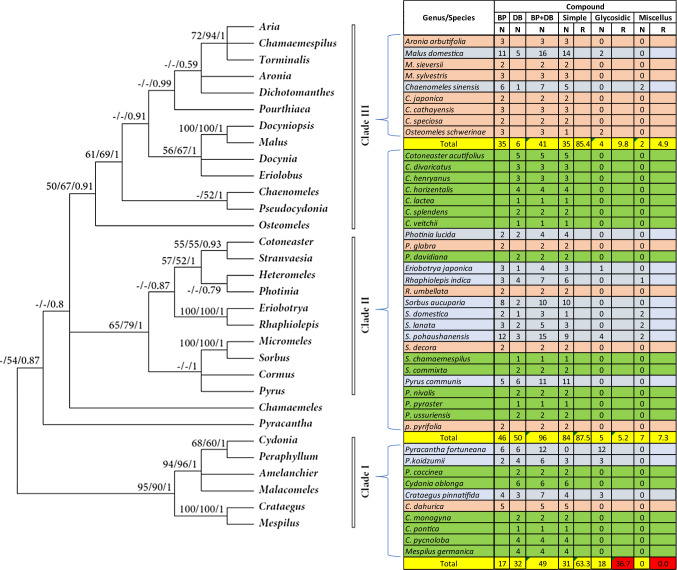
Phylogenetic distribution of biphenyl and dibenzofuran phytoalexins in the Malinae. The phylogenetic tree of the Malinae genera is modified after Sun et al. (2018). Values beside the branches are the bootstrap percentages from maximum parsimony analysis, maximum likelihood analysis, and Bayesian posterior probabilities. “-” indicates a branch collapse in the maximum parsimony and maximum likelihood trees. BP, biphenyl; DB, dibenzofuran; N, number of phytoalexins; R, ratio of the number of phytoalexins in a class to the total number of phytoalexins; Shaded in orange, plants producing biphenyls only; Green, plants producing dibenzofurans only; Grey, plants producing both biphenyls and dibenzofurans; Red, values indicating clear differences among the three clades

As presented in Fig. 1, the chemical structures of biphenyls and dibenzofurans in the Malinae are diverse. They involve simple molecules with hydroxy and/or methoxy substitutions at the aromatic rings of the basic biphenyl and dibenzofuran skeletons (**1–13**, **17–20**, **27, 28**, **33–36**, **44**, **47–55**, **57–63**, **66–70**, **72–76**, **83**, **84**, **86**, **87**; in total 54 structures), *O-*glycosides with glucose and/or rhamnose moieties (**14–16**, **21–26**, **32**, **40–43**, **45**, **46**, **56**, **77–82**; in total 23 compounds), and miscellaneous structures with aliphatic, aromatic, and/or heterocyclic substituents at the aromatic rings of the basic skeletons (**29–31**, **37–39**, **64**, **65**, **71**, **85**; in total 10 structures). These biphenyl and dibenzofuran compounds were detected in 44 species of 14 Malinae genera, which were distributed among three clades in a phylogenetic tree (Fig. 2; Tables 1, 2) (Sun et al. 2018). A detailed analysis indicated that most species in clade III (7 out of 9) contain only biphenyls. In contrast, approximately half of the species in clade II (13 out of 25) and clade I (6 out of 10) form only dibenzofurans. In addition, the types of phytoalexin structures differed between the three clades. In species of clades I, II, and III, simple molecules constituted 63.3%, 87.5%, and 85.4%, respectively. Glycosidic biphenyls and dibenzofurans made up 36.7% in species of clade I, whereas their portions in clades II and III were only 5.2% and 9.8%, respectively. Furthermore, the clade I species did not have any miscellaneous structures which, however, constituted 7.3% and 4.9% in clade II and III species, respectively. The genes for biphenyl and dibenzofuran biosynthetic enzymes are thus differentially regulated in the Malinae species.

**Table 1:** Distribution and function of biphenyls in the rosaceous subtribe Malinae

Genus/Species	Biphenyl		Plant part	References
	Phytoalexin	Phytoalexin?		
*Aronia*				
*A. arbutifolia*	3, 7, 9		SW	Kokubun and Harborne (1995)
*Chaenomeles*				
*C. cathayensis*	3, 7, 9		SW	Kokubun and Harborne (1995)
*C. japonica*	7, 9		SW	Kokubun and Harborne (1995)
*C. sinensis*		3, 6, 7, 28, 29, 31	T	Kim et al. (2016)
*C. speciosa*		3, 7, 28–30	T,F	Suh et al. (2017); Huang et al. (2018); Wang et al. (2021)
*Crataegus*				
*C. dahurica*		7, 33–36	F	Wang et al. (2020)
*C. pinnatifida*		9, 40–43	L	Chen et al. (2006); Huang et al. (2012); Song et al. (2012)
*Eriobotrya*				
*E. japonica*	3, 6	43	L, S	Watanabe et al. (1982); Morita et al. (1983); Miyakodo et al. (1985); Morita and Nonaka (2003); Li and Xuan (2006)
*Malus*				
*M. domestica*	1–3, 6, 7, 9–13, 23		CC, R, SW, S, SM	Kokubun and Harborne (1995); Borejsza-Wysocki et al. (1999); Chizzali et al. (2012a, b); Chizzali et al. (2013); Sarkate et al. (2018); Sarkate et al. (2019); Busnena et al. (2021)
*M. sieversii*	3, 7,		SW	Kokubun and Harborne (1995)
*M. sylvestris*	3, 7, 9		SW	Kokubun and Harborne (1995)
*Osteomeles*				
*O. schwerinae*		44–46	L,T	Lee et al. (2015)
*Photinia*				
*P. glabra*	7, 9		L	Widyastuti et al. (1992)
*P. lucida*		3, 7	SM	Chen et al. (2009)
*Pyracantha*				
*P. fortuneana*		21–26	F	Dai et al. (2006, 2009)
*P. koidzumii*		6, 32	F	Lin et al. (2015)
*Pyrus*				
*P. communis*	1–4, 6		S	Chizzali et al. (2012a, b); Chizzali et al. (2016)
*P. pyrifolia*	2, 3		CC	Saini et al. (2019, 2020)
*Rhaphiolepis*				
*R. indica*		1, 11, 27	R	Lin et al. (2010)
*R. umbellata*	8, 9		L	Watanabe et al. (1990); Widyastuti et al. (1991a, 1991b)
*Sorbus*				
*S. aucuparia*	2, 3, 5–7, 9, 10, 12		CC, HW, L, SW	Erdtman et al. (1961, 1963); Kokubun and Harborne (1994, (1995); Kokubun et al. (1995a); Liu et al. (2004); Hüttner et al. (2010); Khalil et al. (2013); Coyne et al. (2014); Jia et al. (2018)
*S. decora*		3, 7	HW	Narasimhachari and Rudloff (1962)
*S. domestica*		38, 39	F	Termentzi et al. (2009)
*S. lanata*		3, 7, 37	STW	Uddin et al. (2013)
*S. pohaushanensis*	2–4, 6, 14–20, 23		CC, L	Zhou et al. (2016); Gao et al. (2021); Song et al. (2021)

Phytoalexin? Role as phytoalexin not yet studied

*B* bark; *CC* cell culture; *F* fruit; *HW* heartwood; *L* leaf; *R* root; *S* shoot; *SM* stem; *STW* stem wood; *SW* sapwood; *T* twig

**Table 2:** Distribution and function of dibenzofurans in the rosaceous subtribe Malinae

Genus/Species	Dibenzofuran		Plant part	References
	Phytoalexin	Phytoalexin?		
*Chaenomeles*				
*C. sinensis*	51		T, SW	Kokubun and Harborne (1995); Kim et al. (2016)
*Cotoneaster*				
*C. acutifolius*	45–51		SW	Kokubun et al. (1995b); Kokubun and Harborne (1995)
*C. divaricatus*	47–49		SW	Kokubun et al. (1995b); Kokubun and Harborne (1995)
*C. henryanus*	47–49		SW	Kokubun et al. (1995b); Kokubun and Harborne (1995)
*C. horizentalis*	47–49, 51		SW	Kokubun et al. (1995b); Kokubun and Harborne (1995)
*C. lactea*	47		SW	Burden et al. (1984); Kokubun and Harborne (1995)
*C. splendens*	47, 49		SW	Kokubun et al. (1995b); Kokubun and Harborne (1995)
*C. veitchii*	49		SW	Kokubun and Harborne (1995)
*Crataegus*				
*C. monogyna*	47, 49		B, SW	Kokubun et al. (1995c); Kokubun and Harborne (1995)
*C. pinnatifida*		48, 86, 87	F, R	Deng et al. (2006); Zhao et al. (2019)
*C. pontica*		52	B, SW	Kokubun et al. (1995c)
*C. pycnoloba*		57, 61, 83, 84	AP	Agalou et al. (2018)
*Cydonia*				
*C. oblonga*	50–52	73–75	SW	Kokubun and Harborne (1995); Wei et al. (2020)
*Eriobotrya*				
*E. japonica*	53		L	Miyakodo et al. (1985); Morita and Nonaka (2003)
*Malus*				
*M. domestica*	53, 54, 56, 66, 67		CC, SM, R	Hrazdina et al. (1997); Chizzali et al. (2012a, b); Chizzali et al. (2013); Zhu et al. (2014); Sarkate et al. (2018); Sarkate et al. (2019); Busnena et al. (2021)
*Mespilus*				
*M. germanica*	47, 61–63		SW	Kokubun et al. (1995d); Kokubun and Harborne (1995)
*Photinia*				
*P. davidiana*	53, 57		SW	Kokubun et al. (1995c); Kokubun and Harborne (1995)
*P. lucida*		53, 54	SM	Chen et al. (2009)
*Pyracantha*				
*P. coccinea*	53, 55		SW	Kokubun et al. (1995c); Kokubun and Harborne (1995)
*P. fortuneana*		77–82	F	Dai et al. (2008)
*P. koidzumii*		55, 76, 80, 82	F	Lin et al. (2015)
*Pyrus*				
*P. communis*	52–54, 58–60		SW, S	Kemp et al. (1983); Kemp and Burden (1984); Kokubun and Harborne (1995); Chizzali et al. (2012a, b); Chizzali et al. (2016)
*P. nivalis*	49, 52		SW	(Kokubun and Harborne 1995)
*P. pyraster*	52		SW	Kokubun and Harborne (1995)
*P. ussuriensis*	49, 52		SW	Kokubun and Harborne (1995)
*Rhaphiolepis*				
*R. indica*		68–71	R	Lin et al. (2010)
*Sorbus*				
*S. aucuparia*	53, 54		CC	Hüttner et al. (2010); Khalil et al. (2013); Coyne et al. (2014)
*S. chamaemespilus*	49		SW	Kokubun and Harborne (1995)
*S. commixta*	59, 72		B	Choi et al. (2018)
*S. domestica*	49		SW	Kokubun and Harborne (1995)
*S. lanata*		59, 85	STW	Yousuf et al. (2010); Uddin et al. (2013)
*S. pohaushanensis*	53, 64, 65		CC	Gao et al. (2021)

Phytoaleixn? Role as phytoalexin not yet studied

*B* bark; *CC* cell culture; *F* fruit; *HW* heartwood; *L* leaf; *R* root; *S* shoot; *SM* stem; *STW* stem wood; *SW* sapwood; *T* twig

The co-occurrence of the two classes of compounds was reported for 13 species of nine Malinae genera, which were distributed among the three clades in the phylogenetic tree: two genera (*Malus* and *Chaenomeles*) in clade III, five genera (*Photinia*, *Eriobotrya*, *Rhaphiolepis*, *Sorbus,* and *Pyrus*) in clade II, and two genera (*Pyracantha* and *Crataegus*) in clade I (Fig. 2; Table 3). In these nine genera, co-occurrence of biphenyls and dibenzofurans was not found in all species. For example, *Sorbus aucuparia*, *S. domestica*, *S. lanata*, and *S. pohaushanensis* contained both classes of phytoalexins, whereas *S. commixta* and *S. chamaemespilus* had only dibenzofurans and *S. decora* had only biphenyls. On the other hand, two genera in clade III (*Aronia* and *Osteomeles*) contained only biphenyls, whereas one genus in clade II (*Cotoneaster*) and two genera in clade I (*Cydonia* and *Mespilus*) contained only dibenzofurans.

**Table 3:**
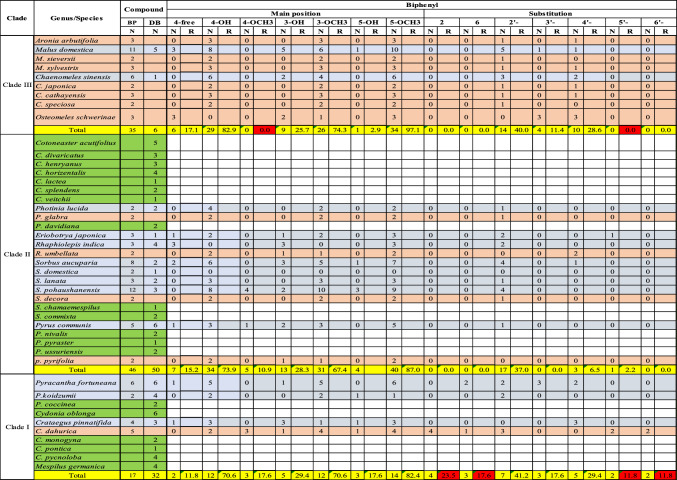

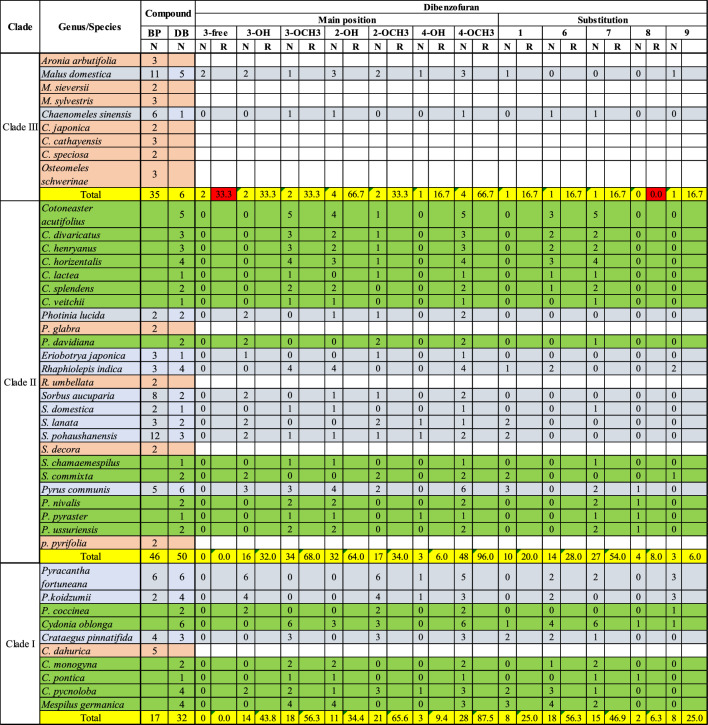
Phylogenetic analysis of the substitution patterns of basic biphenyl and dibenzofuran skeletons

Shaded in orange, plants producing biphenyls only; Green, plants producing dibenzofurans only; Grey, plants producing both biphenyls and dibenzofurans; Red, substitutions with clear differences between the three clades

*BP* biphenyl; *DB* dibenzofuran; *N* number; *R* ratio

Likewise, the substitution patterns of the biphenyl and dibenzofuran basic skeletons varied among the three clades of the Malinae genera (Table 3). In the clade III species, none of the 35 biphenyls were substituted by methoxy (-OCH3) at C-4, whereas 10.9% and 17.6% of the biphenyls in clade II and I, respectively, had a methoxy moiety at C-4. In addition, 23.5%, 17.6%, 11.8%, and 11.8% of the biphenyls in species of clade I carried substitutions at positions C-2, C-6, C-5ʹ, and C-6ʹ, respectively. On the other side, no substitutions at these positions were observed for biphenyls of clade II and III species, except for 2.2% of clade II biphenyls, which had a substitution at C-5ʹ. For dibenzofurans, 33.3% of clade III species lacked a substitution at C-3 (equal to C-4 in biphenyls), whereas the C-3 position of all dibenzofurans was substituted in clades I and II. Furthermore, the C-8 position (equal to C-5ʹ in biphenyls) was free of substitution in clade III species only. Regiospecific substitutions on the basic skeletons are accomplished by modifying enzymes, such as hydroxylases, methyltransferases, and glycosyltransferases. The diversity within the individual enzyme classes is commonly due to gene duplication events. Obviously, the Malinae species differ in array and activity of genes for modifying enzymes.

The most widely distributed biphenyls are aucuparin (**3**), 2'-methoxyaucuparin (**7**), and 4'-methoxyaucuparin (**9**) (Table 1). Aucuparin (**3**) and 2'-methoxyaucuparin (**7**) were isolated for the first time from the healthy heartwood of *S. aucuparia*. Thereafter, aucuparin (**3**) was detected in fourteen species belonging to seven genera (*Aronia, Chaenomeles, Eriobotrya, Malus, Photinia, Pyrus, Sorbus*). Its role as phytoalexin was disclosed in ten species. 2'-Methoxyaucuparin (**7**) was isolated from fourteen species belonging to six genera (*Aronia, Chaenomeles, Crataegus, Malus, Photinia* and *Sorbus*). Its function as phytoalexin was observed in eight species. Furthermore, 4'-methoxyaucuparin (**9**) was detected as phytoalexin in eight species belonging to six genera (*Aronia, Chaenomeles, Malus, Photinia, Rhaphiolepis, Sorbus*). Interestingly, the biphenyls **5**, **8**, **13–22**, **24–27**, and **30–46** were exclusively detected in individual species (Table 1).

Regarding dibenzofurans, *α*-cotonefuran (**47**), *γ*-cotonefuran (**49**), and eriobofuran (**53**) are the most widely distributed compounds (Table 2). *γ*-Cotonfuran (**49**) was detected as phytoalexin in eleven species belonging to four genera (*Cotoneaster, Crataegus, Pyrus, Sorbus*). *α*-Cotonefuran (**47**) was found as phytoalexin in eight species belonging to three genera (*Cotoneaster, Crataegus, Mespilus*). Moreover, eriobofuran (**53**) was isolated from eight species belonging to six genera (*Eriobotrya, Malus, Photinia, Pyracantha, Pyrus, Sorbus*). However, its role as phytoalexin was not confirmed in *Photinia lucida*. Interestingly, the dibenzofurans **56**, **58**, **60**, **62–79**, **81**, and **83–87** were exclusively isolated from individual species.

The function of biphenyls and dibenzofurans as phytoalexins was reported for a total of 31 out of 44 species in the subtribe Malinae (Tables 1 and 2). Eight species form only biphenyls (*Aronia arbutifolia, Chaenomeles cathayensis, Chaenomeles japonica, Malus sieversii, Malus sylvestris, Photinia glabra, Pyrus pyrifolia, Rhaphiolepis umbellata*), sixteen species contain only dibenzofurans (*Cotoneaster acutifolius, Cotoneaster divaricatus, Cotoneaster henryanus, Cotoneaster horizontalis, Cotoneaster lacteal, Cotoneaster splendens, Cotoneaster veitchii, Crataegus monogyna, C. oblonga, Mespilus germanica, Photinia davidiana, Pyracantha. coccinea, Pyrus nivalis, Pyrus pyraster, Pyrus ussuriensis, S. chamaemespilus*), and seven species have both biphenyls and dibenzofurans (*Eriobotrya japonica, M. domestica, P. communis, S. aucuparia, S. pohaushanensis*), including two species (*Chaenomeles sinensis*, *S. domestica*), in which the role of the biphenyls as phytoalexins remains open. In 13 out of 44 species in the subtribe Malinae (Tables 1 and 2), neither biphenyls nor dibenzofurans were examined for their role as phytoalexins. Four species produce only biphenyls (*Chaenomeles speciosa, Crataegus dahurica, Osteomeles schwerinae, S. decora*), three species have only dibenzofurans (*Crataegus pontica, Crataegus pycnoloba, S. commixta*), and six species form both classes of compounds (*Crataegus pinnatifida, Photinia lucida*, *Pyracantha fortuneana, Pyracantha koidzumii, Rhaphiolepis indica*, *S. lanata*).

It is noteworthy that 21 biphenyls (**1–20** and **23**) and 21 dibenzofurans (**47–67**) were reported to be produced as phytoalexins in response to stress factors (Tables 1 and 2). In contrast, the phytoalexin function of 25 biphenyls (**21**, **22**, **24–46**) and 20 dibenzofurans (**68–87**) has not yet been explored. Curiously, 2,8-dihydroxy-3,4,7-trimethoxydibenzofuran (**52**) was produced as phytoalexin in five species (*C. oblonga, P. communis, P. nivalis, P. pyraster, P. ussuriensis*) but constitutively formed in *C. potinica*.

Taken together, biphenyls and dibenzofurans are not only diverse in their chemical structures but also variable in their abundance among the species of the subtribe Malinae. Some species accumulate a wide array of biphenyls and dibenzofurans, whereas others form only a few compounds. Furthermore, different parts of the producing plants contain the phytoalexins. Some compounds were isolated from the roots of a limited number of species, whereas the majority of phytoalexins were found in the aerial parts. Most biphenyls were detected in sapwood, leaf, and fruit, whereas most dibenzofurans were restricted to the sapwood. Functionally, nearly half of the reported biphenyls (21 out of 46) and dibenzofurans (21 out of 41) were demonstrated to act as phytoalexins, indicating that these two classes of compounds are the inducible defense substances of the Malinae, although residual compounds still need to be explored for their phytoalexin role. The clade-related distribution of biphenyls and dibenzofurans and the substitution patterns of their basic skeletons are interesting issues for chemotaxonomy and evolution of the Malinae. However, we must bear in mind that the number of species explored for the formation of biphenyls and dibenzofurans (44 species) is still small compared to the total number of species present in this subtribe (1000 species). With an increasing number of species investigated, more structures of biphenyls and dibenzofurans will be identified, which in turn will allow clear-cut chemotaxonomic conclusions.

## Biosynthesis of biphenyls and dibenzofurans

**Fig. 3:**
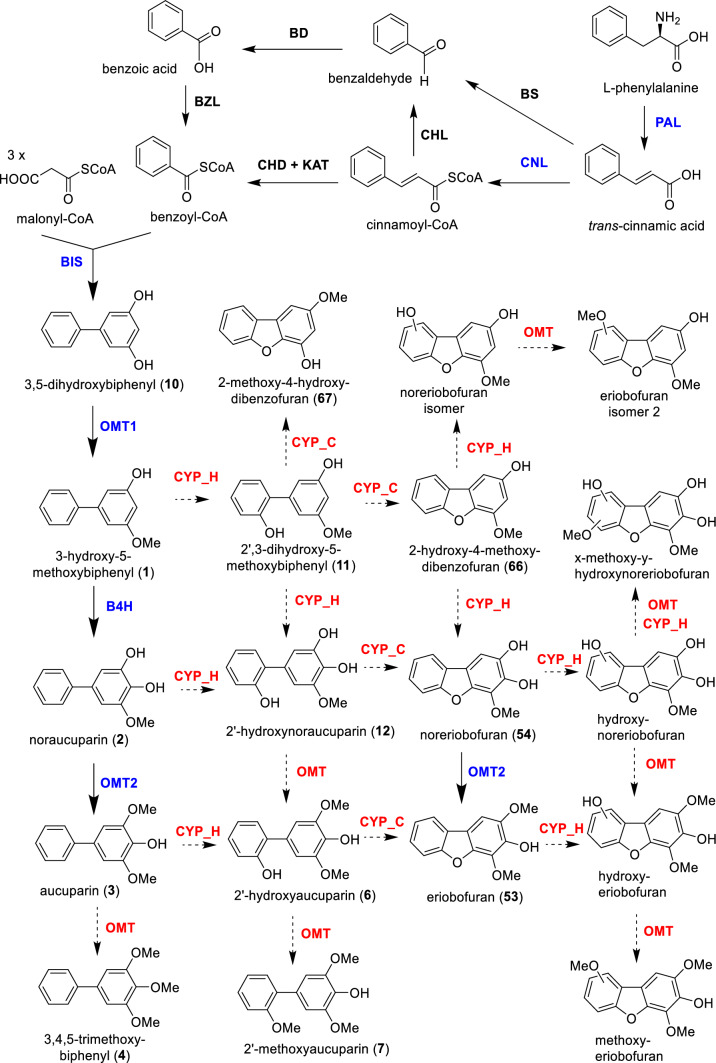
Biosynthesis of biphenyls and dibenzofurans. Solid arrows with blue enzyme names indicate reactions established at the gene level; solid arrows with black enzyme names indicate reactions demonstrated at the biochemical level; broken arrows with red enzyme names indicate proposed reactions. *B4H* biphenyl 4-hydroxylase; *BD* benzaldehyde dehydrogenase; *BIS* biphenyl synthase; *BS* benzaldehyde synthase; *BZL* benzoate-CoA ligase; *CHD* cinnamoyl-CoA hydratase/dehydrogenase; *CHL* cinnamoyl-CoA hydratase/lyase; *CNL* cinnamoyl-CoA ligase; *CYP_H* cytochrome P450 hydroxylase; *CYP_C* cytochrome P450 cyclase; *KAT* ketoacylthiolase; *OMT* *O*-methyltransferase; *PAL* phenylalanine ammonia lyase (Abd El-Mawla and Beerhues 2002; Liu et al. 2004, 2007; Hüttner et al. 2010; Chizzali et al. 2012a, b; Khalil et al. 2013, 2015; Sircar et al. 2015; Saini et al. 2017, 2019, 2020; Sarkate et al. 2019; Teotia et al. 2019; Busnena et al. 2021; Song et al. 2021)

The biosynthetic pathway of biphenyl and dibenzofuran phytoalexins is only fragmentarily known. A completely elucidated route at both the biochemical and molecular levels is the biosynthesis of the most common biphenyl, aucuparin (**3**) in cell cultures of *S. aucuparia* (Fig. 3). Biphenyl synthase (BIS), a type III polyketide synthase, catalyzes the first committed step of the pathway to form 3,5-dihydroxybiphenyl (**10**) (Liu et al. 2004, 2007). *O*-Methyltransferase 1 (OMT1) then catalyzes methylation of 3,5-dihydroxybiphenyl (**10**) to form 3-hydroxy-5-methoxybiphenyl (**1**), which in turn is hydroxylated at the C-4 position by a cytochrome P450 enzyme, biphenyl 4-hydroxylase (B4H), to yield noraucuparin (**2**) (Khalil et al. 2013, 2015; Sircar et al. 2015). The hydroxy group at C-3 of noraucuparin (**2**) is subsequently methylated by *O*-methyltransferase 2 (OMT2) to form aucuparin (**3**). Notably, the counterparts of these aucuparin biosynthetic genes present in *M. domestica* were also cloned and functionally characterized (Chizzali et al. 2012b; Sircar et al. 2015; Sarkate et al. 2019).

Based on the structures of the isolated biphenyls, 3,5-dihydroxybiphenyl (**10**) can undergo single or multiple hydroxylation and methylation steps at the carbons 2, 4, 2ʹ, 3ʹ, and 4ʹ. However, detailed biochemical and molecular studies are still needed to elucidate these individual steps. In *S. pohaushanensis,* the presence of 3,4,5-trimethoxybiphenyl (**4**) (Song et al. 2021) points to another *O*-methyltransferase which methylates the 4-hydroxy group of aucuparin (**3**), however, this step is also open at the enzyme and gene levels.

The conversion of biphenyls to dibenzofurans has not yet been confirmed experimentally. This intramolecular cyclization requires biphenyl 2ʹ-hydroxylation. The resulting 2ʹ-hydroxylated intermediate does not originate from salicoyl-CoA as a BIS starter substrate (Liu et al. 2010; Chizzali and Beerhues 2012). Upon salicoyl-CoA usage, all the known BIS enzymes release 4-hydroxycoumarin after a single condensation with malonyl-CoA rather than 2ʹ,3,5-trihydroxybiphenyl after three decarboxylative condensations. In *S. aucuparia*, the only isolated 2ʹ-hydroxylated biphenyl was 2ʹ-hydroxyaucuparin (**6**) (Hüttner et al. 2010) and no dibenzofuran lacking substitution at C-3 (corresponding to C-4 in biphenyls) was detected. These facts argue against the transition of 3,5-dihydroxybiphenyl (**10)** and 3-hydroxy-5-methoxybiphenyl (**1**) to the corresponding dibenzofurans. Since noreriobofuran (**54**) was detected in *S. aucuparia* cell cultures, noraucuparin (**2**) was believed to be the metabolic branch point, from which biphenyls derive on one side and dibenzofurans on the other side (Khalil et al. 2015). However, 2ʹ,3-dihydroxy-5-methoxybiphenyl (**11**) and 2ʹ-hydroxy-noraucuparin (**12**) have recently been detected in roots and medium of in vitro cultured and yeast-extract-treated plants of the apple rootstock M26 (Busnena et al. 2021). The profile of biphenyls in *M. domestica* indicates that biphenyl 2ʹ-hydroxylation can take place before hydroxylation at C-4. Interestingly, intramolecular cyclization of 2ʹ,3-dihydroxy-5-methoxybiphenyl (**11**) forms 2-hydroxy-4-methoxydibenzofuran (**66**), which was found for the first time in apple (Weiß et al. 2017a). In fact, 2-hydroxy-4-methoxydibenzofuran (**66**) is the predominant dibenzofuran in nearly all apple root samples studied (Reim et al. 2020; Rohr et al. 2020; Balbín-Suárez et al. 2021; Busnena et al. 2021). Thus, 3-Hydroxy-5-methoxybiphenyl (**1**) seems to be the target of 2ʹ-hydroxylation and the resulting product, 2ʹ,3-dihydroxy-5-methoxybiphenyl (**11**), seems to be the branch point of the biphenyl and the dibenzofuran biosynthetic routes in apple (Fig. 3). The cyclization of 2ʹ,3-dihydroxy-5-methoxybiphenyl (**11**) to form 2-hydroxy-4-methoxydibenzofuran (**66**) is proposed to be a major biosynthetic step in apple which, however, is absent from *S. aucuparia.* Another main phytoalexin in apple is the eriobofuran isomer 2 (Busnena et al. 2021), which is supposed to be synthesized through hydroxylation of 2-hydroxy-4-methoxydibenzofuran (**66**).

**Fig. 4:**
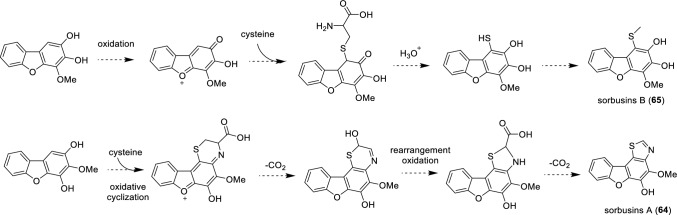
Proposed biosynthetic pathway of sulfur and nitrogen-containing dibenzofurans (Gao et al. 2021)

The formation of *O*-glycosides of biphenyl and dibenzofuran compounds is likely to be catalyzed by glycosyltransferase (GTF) enzymes. Although no public report about biphenyl and dibenzofuran GTFs was published, a phytoalexin GTF from *S. pohaushanensis* was described in two patents (Huang et al. 2017a, b). The biosynthesis of sorbusin A (**64**) and sorbusin B (**65**), which are sulfur and nitrogen-containing dibenzofurans, was proposed to be a condensation of cysteine with the oxidated dibenzofurans, followed by a series of reactions like cyclization, decarboxylation, re-arrangement, and methylation (Fig. 4) (Gao et al. 2021). However, this hypothesis remains to be proven experimentally. Chaenomin A (**29**), chaenomin B (**30**), berbekorin A (**31**), and sorbalanin (**85**) share a common component (4-hydroxyphenylglycerol), which is probably derived from the phenylpropanoid metabolism and attached to the biphenyl and dibenzofuran skeletons by either the 4-hydroxy group or the middle hydroxy group of glycerol. Biphenyls **38** and **39** lack the typical substitution pattern of 3,5-dihydroxybiphenyl. Instead, we propose that they arise from the C–C coupling of two phenylpropanoid units, which undergo esterification with either hexanol or heptanol and hydroxylation.

The biosynthesis of benzoyl-CoA, the starter substrate for BIS besides malonyl-CoA as extender, is a branch pathway of phenylpropanoid metabolism (Fig. 3). Cinnamic acid is formed from the aromatic amino acid L-phenylalanine by phenylalanine ammonium lyase (PAL). In the Malinae species, cinnamic acid is converted to benzoic acid by two pathways. The first pathway is CoA-independent and non-β-oxidative, which involves benzaldehyde synthase (BS) and benzaldehyde dehydrogenase (BD). This pathway was demonstrated at the biochemical level in *P. pyrifolia* (Saini et al. 2017, 2019)*.* Alternatively, cinnamic acid is converted to cinnamoyl-CoA by cinnamoyl-CoA ligase (CNL), which is further converted to benzaldehyde through the CoA-dependent and non-β-oxidative pathway. Although cinnamoyl-CoA formation was demonstrated in apple (Teotia et al. 2019), the conversion of cinnamoyl-CoA to benzaldehyde by cinnamoyl-CoA hydratase/lyase (CHL) was only detected in *Hypericum androsaemum* (Abd El-Mawla and Beerhues 2002)*.* Benzoyl-CoA ligase (BZL) activity has recently been detected at the biochemical level in cell cultures of *P. pyrifolia* (Saini et al. 2020).

The above findings indicate that a number of reactions related to biphenyl and dibenzofuran biosynthesis were elucidated. The enzymes that catalyze these reactions and the genes that encode the responsible enzymes were investigated. However, progress was mainly achieved with biphenyl biosynthesis, whereas dibenzofuran formation remains largely obscure. This is particularly true for the central biosynthetic steps of dibenzofuran biosynthesis, i.e. 2ʹ-hydroxylation and intramolecular cyclization to yield the tricyclic scaffold. The efforts have to be made to fully clarify the formation of the metabolic grid of biphenyl and dibenzofuran compounds.

## Induction of phytoalexin formation by biotic and abiotic stimuli

Generally, phytoalexins formation is induced by biotic and abiotic factors. Abiotic inducers are not derived from biotic sources. They are stress conditions like extremely high or low temperatures, plant wounding, or exposure to UV light and heavy metals (Moesta and Grisebach 1980; Kokubun and Harborne 1994; Pedras et al. 2007). Biotic inducers refer to either intact microorganisms or extracts and molecules derived thereof (Darvill and Albersheim 1984; Khan et al. 2021). Only three studies reported phytoalexin formation in Malinae species upon elicitation by abiotic inducers, mainly heavy metals (Table 4). Mercuric chloride induced the formation of 2'-methoxyaucuparin (**7**) and 4'-methoxyaucuparin (**9**) in the leaves of *P. glabra*. It also stimulated the formation of rhaphiolepsin (**8**) and 4'-methoxyaucuparin (**9**) in leaves of *Rhaphiolepis umbellata*. Aucuparin (**3**) was induced in the leaves of *S. aucuparia* upon exposure to copper sulfate solution. Heavy metals as inducers are of ecological relevance because plants can be exposed to various heavy metals as pollutants in air, water, and soil.

**Table 4:** Induction of biphenyl and dibenzofuran phytoalexins in the rosaceous subtribe Malinae

Inducer	Species	Phytoalexin	Plant part	References
Abiotic inducer				
Mercuric chloride	*P. glabra*	7, 9	L	Widyastuti et al. (1992)
	*R. umbellata*	8, 9	L	Watanabe et al. (1990), Widyastuti et al. (1991a)
Copper sulfate	*S. aucuparia*	3	L	Kokubun and Harborn (1994)
Fungi				
*Alternaria tenuissi*	*S. pohaushanensis*	2-4, 6, 17-20	L	Song et al. (2021)
*Ceratocystis ulmi*	*C. lactea*	47	SW	Burden et al. (1984); Kokubun and Harborne (1995)
*Chondrostereum purpureum pouzar*	*P. communis*	58-60	SW	Kemp et al. (1983); Kemp and Burden (1984)
*Colletotrichum lindemuthianum*	*E. japonica*	3, 6	S	Watanabe et al. (1982); Miyakodo et al. (1985)
*Entomosporium eriobotryae*	*E. japonica*	53	L	Miyakodo et al. (1985)
*Entomosporium mespili*	*P. glabra*	7, 9	L	Widyastuti et al. (1992)
	*R. umbellata*	8	L	Watanabe et al. (1990); Widyastuti et al. (1991b)
*Nectria cinnabarina*	*C. acutifolius*	47-51	SW	Kokubun et al. (1995b); Kokubun and Harborne (1995)
	*C. divaricatus*	47-49	SW	Kokubun et al. (1995b); Kokubun and Harborne (1995)
	*C. henryanus*	47-49	SW	Kokubun et al. (1995b); Kokubun and Harborne (1995)
	*C. horizentalis*	47-49, 51	SW	Kokubun et al. (1995b); Kokubun and Harborne (1995)
	*C. splendens*	47, 49	SW	Kokubun et al. (1995b); Kokubun and Harborne (1995)
	*M. germanica*	47, 61-63	SW	Kokubun et al. (1995d); Kokubun and Harborne (1995)
	*P. davidiana*	53, 57	SW	Kokubun et al. 1995c; Kokubun and Harborne (1995)
	*S. aucuparia*	3, 5-7, 9	SW	Kokubun et al. (1995a); Kokubun and Harborne (1995)
*Nectria galligena *and *Phomopsis perniciosa*	*A. arbutifolia*	3, 7, 9	SW	Kokubun and Harborne (1995)
	*C. cathayensis*	3, 7, 9	SW	Kokubun and Harborne (1995)
	*C. japonica*	7, 9	SW	Kokubun and Harborne (1995)
	*C. sinensis*	51	SW	Kokubun and Harborne (1995)
	*C. lactea*	47	SW	Burden et al. (1984); Kokubun and Harborne (1995)
	*C. veitchii*	49	SW	Kokubun and Harborne (1995)
	*C. monogyna*	47, 49	B, SW	Kokubun et al. (1995c); Kokubun and Harborne (1995)
	*C. oblonga*	50-52	SW	Kokubun and Harborne (1995)
	*M. sieversii*	3, 7	SW	Kokubun and Harborne (1995)
	*M. silvestris*	3, 7, 9	SW	Kokubun and Harborne (1995)
	*P. davidiana*	53, 57	SW	Kokubun et al. (1995c); Kokubun and Harborne (1995)
	*P. coccinea*	53, 55	SW	Kokubun et al. (1995c); Kokubun and Harborne (1995)
	*P. communis*	52, 59, 60	SW	Kokubun and Harborne (1995)
	*P. nivalis*	49, 52	SW	Kokubun and Harborne (1995)
	*P. pyraster*	52	SW	Kokubun and Harborne (1995)
	*P. ussuriensis*	49, 52	SW	Kokubun and Harborne (1995)
	*S. aucuparia*	3, 5-7, 9	SW	Kokubun et al. (1995a); Kokubun and Harborne (1995)
	*S. chamaemespilus*	49	SW	Kokubun and Harborne (1995)
	*S. domestica*	49	SW	Kokubun and Harborne (1995)
*Phomopsis perniciosa*	*M. domestica*	3, 7	SW	Kokubun and Harborne (1995)
*Ventoria inaequalis*	*M. domestica*	2, 3, 53	CC	Sarkate et al. (2018, 2019)
	*S. aucuparia*	2, 3, 6, 53, 54	CC	Hüntter et al. (2010); Khalil et al. (2013)
Bacteria				
*Erwina amylovora*	*M. domestica*	1-3, 6, 53, 54	SM	Chizzali et al. (2012a; b, 2013); Coyne et al. (2014)
	*P. communis*	1-4, 6, 53, 54	SM	Chizzali et al. (2012a; b, 2016)
	*S. aucuparia*	2, 3, 6, 53, 54	CC	Hüntter et al. (2010); Coyne et al. (2014)
*Pseudomonas syringae pv. eriobotryae*	*E. japonica*	3, 53	L	Morita et al. (1983); Morita and Nonaka (2003)
*Pseudomonas syringae pv. tabaci*	*E. japonica*	3	L	Morita et al. (1983)
Bio-extracts and molecules				
Chitosan	*S. aucuparia*	2, 3, 6, 53, 54	CC	Hüntter et al. (2010)
Harpin protein crude extract	*S. aucuparia*	2	CC	Jia et al. (2018)
Yeast extract	*M. domestica*	1-3, 6, 7, 9-13, 23, 53, 54, 56, 66, 67	CC	Hrazdina et al. (1997); Borejsza-Wysocki et al. (1999); Busnena et al. (2021)
	*P. pyrifolia*	2, 3	SW	(Saini et al. 2019, 2020)
	*S. aucuparia*	2, 3, 6, 53, 54	CC	Liu et al. (2004); Hüntter et al. (2010); Qiu et al. (2012)
	*S. pohaushanensis*	2, 3, 6, 14-16, 23, 53, 64, 65	CC	Zhou et al. (2016); Gao et al. (2021)
Diseased soil				
Apple replant-diseased soil	*M. domestica*	1-3, 6, 9-11, 53, 54, 66	R	Busnena et al. (2021)

*B* bark; *CC* cell culture; *L* leaf; *R* root; *S* shoot; *SM* stem; *SW* sapwood

In contrast to the few reports of abiotic effects, many research papers documented the formation of phytoalexins in Malinae species upon induction by biotic inducers, i.e. phytopathogens. Included are also yeast extract, harpin protein crude extract (Hpa1), and chitosan but these inducers lack ecological relevance for phytoalexins induction in plants. Nonetheless, they were used to study the phytoalexins profile of some species, mainly under cell culture conditions, aiming at understanding the structural diversity and biosynthetic route of the induced phytoalexins. In fact, fungal attack is considered a major inducer of biphenyl and dibenzofuran phytoalexins in the Malinae. Ten phytopathogenic fungal species were reported to initiate the formation of phytoalexins in this subtribe (Table 4). *Nectria galligena* and *Phomopsis perniciosa* (canker-causative agents in apple and pear) stimulated the production of the phytoalexins **3**, **5–7**,** 9**,**47, 49–53**, **55**, **57**, **59**, **60** in the sapwood of 20 plant species. Notably, they induced the formation of either biphenyls (in seven plant species) or dibenzofurans (in 13 plant species). In addition, *Nectria cinnabarina* (coral spot and canker-causative agent) induced the formation of the biphenyls **3**, **5–7**, and **9** in the sapwood of *S. aucuparia* and the dibenzofurans **47- 51**, **53**, **57**, and **61–63** in the sapwood of seven other plant species*.* Interestingly*, Venturia inaequalis* (scab-causative agent) was the only fungal species to induce the formation of both biphenyls and dibenzofurans in cell cultures of apple (**2**,** 3**, **53**) and *S. aucuparia* (**2**,** 3**, **6**, **53**, **54**).

Besides fungi, only three pathogenic bacteria were reported to induce the formation of biphenyl and dibenzofuran phytoalexins in the Malinae (Table 4). *Erwinia amylovora* (fire blight disease-causing bacterium) stimulated the formation of the phytoalexins **2**, **3**, **6**, **53**, and **54** in apple*, P. communis*, and *S. aucuparia*. It also elicited the formation of 3-hydroxy-5-methoxybiphenyl (**1**) in both apple and pear, whereas 3,4,5-trimethoxybiphenyl (**4**) was only found in *P. communis*. Moreover, *Pseudomonas syringae* pv. tabaci (wildfire disease-causative agent) stimulated leaves of *E. japonica* to produce aucuparin (**3**), whereas *Pseudomonas syringae* pv. eriobotryae (loquat stem canker-causative agent) additionally induced eriobofuran (**53**) formation. Yeast extract elicited an extensive accumulation of biphenyl and dibenzofuran phytoalexins. It differentially induced the production of the phytoalexins **1–3**,** 6**,** 7**, **9–16**, **23**, **53**, **54**, **56**, and **64–67** in four species (*M. domestica, S. aucuparia, S. pouhaushanensis, P. pyrifolia*). In *S. aucuparia* cell cultures, Harpin protein crude extract (*Hpa1*) induced the formation of noraucuparin (**2**), while chitosan, a polysaccharide derived from chitin, induced the production of the phytoalexins **2**,** 3**,** 6**, **53**, and **54** (Table 4).

As described above, phytopathogens are the main inducers of phytoalexin biosynthesis in the Malinae. They stimulated the formation of biphenyls and dibenzofurans differentially, in terms of identity, number, and chemical class. The differences are due to the nature of the inducer, the induction mechanism, the response of the individual plant species, and the type of the plant system used, e.g. undifferentiated cell cultures vs. highly differentiated intact plants. The role of individual phytoalexins and especially their synergism in pathogen defense largely remains to be investigated.

## Apple replant-diseased soil as an in vivo model for phytoalexins induction

Replant disease is a soil-borne disease in orchards and tree nurseries. It affects many economically important species belonging to the Rosaceae, such as apple, cherry, and peach. Apple replant disease (ARD) was reported to suppress the vegetative and generative performance of apple cultivars by up to 50% and to delay the bearing of fruits on trees by 2–3 years (Mai and Abawi 1978; Winkelmann et al. 2019; Cavael et al. 2020). Although the etiology of ARD is still not fully understood, biotic factors were proven to be the major causal agents. Indeed, there are many reports of an altered microbiome, i.e., changes in the fungal and bacterial communities in ARD-affected soil, which may exacerbate or ameliorate the effects of phytopathogens. There is increasing evidence that multiple soil-borne detrimental fungi (*Rhizoctonia* sp., *Fusarium* sp., *Cylindrocarpon* sp., and other *Nectriaceae*), oomycetes (*Phytophthora* sp. and *Pythium* sp.), and nematodes (*Pratylenchus penetrans*) form a disease complex of variable species (Kanfra et al. 2018; Manici et al. 2018; Radl et al. 2019; Balbín-Suárez et al. 2020).

**Fig. 5:**
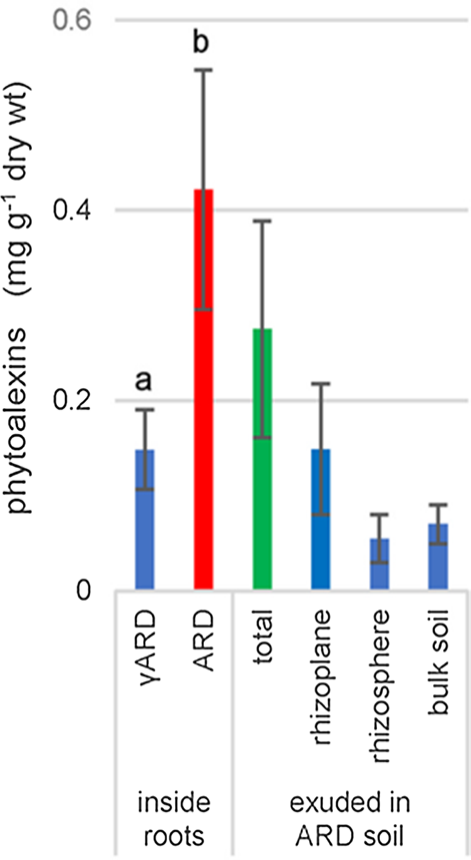
Total biphenyl and dibenzofuran phytoalexins inside and outside roots of the apple rootstock M26 cultivated in either gamma-irradiated (γARD) or untreated ARD soils. Total is the sum of phytoalexin amounts detected in rhizoplane, rhizosphere, and bulk soil (adapted from Busnena et al. 2021)

The symptoms of ARD include general stunting and reduction in biomass. The root system becomes damaged, fragile, and dark-colored. The roots of diseased apple plants contain higher amounts of phenolics and antioxidants than healthy roots (Henfrey et al. 2015; Rohr et al. 2021). Specifically, the roots accumulate higher amounts of biphenyls and dibenzofurans (Kanfra et al. 2018; Reim et al. 2020; Rohr et al. 2020; Busnena et al. 2021). These phytoalexins were detected for the first time in roots of the apple rootstock M26 when cultivated in ARD-affected soil (Weiß et al. 2017a). M26, besides other apple rootstock genotypes like B63 and MAL0595, formed the biphenyls **1**–**3**, **6**, **11** and the dibenzofurans **53**, **54**, **66** (Reim et al. 2020; Rohr et al. 2020; Balbín-Suárez et al. 2021). Recently, we have even detected seven biphenyls (**1–3**,** 6**, **9–11**) and twenty dibenzofurans (**53**, **54**, **66**) plus seventeen tentatively identified dibenzofurans in M26 rootstock upon growth in ARD-affected soil (Busnena et al. 2021). Notably, the phytoalexins were not only accumulated inside the roots but also exuded to the root surface and the surrounding soil, where they exert effects on the microbiome (Fig. 5).

**Fig. 6:**
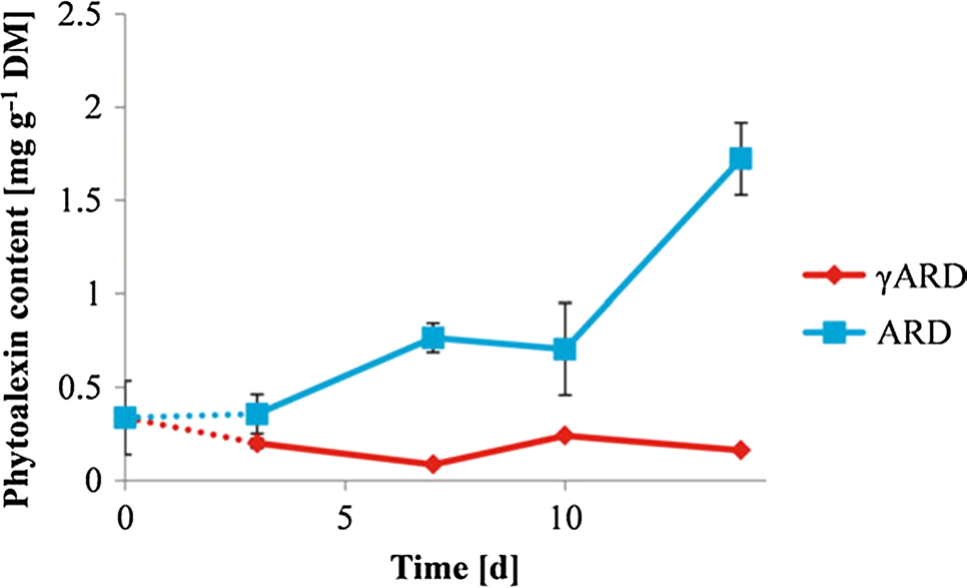
Total biphenyl and dibenzofuran phytoalexins content of roots of the apple rootstock M26 cultivated in either gamma-irradiated (γARD) or untreated ARD soils for 14 days (Weiß et al. 2017a, b)

Genes involved in phytoalexin biosynthesis, such as *biphenyl synthase* (*BIS*) and *biphenyl-4-hydroxylase* (*B4H*), were upregulated in ARD samples as early as three days after planting (Weiß et al. 2017b; Reim et al. 2020; Rohr et al. 2020; Balbín-Suárez et al. 2021). Consequently, the biphenyl and dibenzofuran phytoalexin amounts increased starting from day 3 and the highest amounts were observed in the root samples after 14 days of cultivation (Fig. 6). Furthermore, the genotype-specific *BIS* and *B4H* expression patterns were consistent with the phytoalexin contents measured in roots (Weiß et al. 2017b; Reim et al. 2020). The *BIS* gene appeared to be particularly useful as an early biomarker for ARD because its expression pattern well correlated with the phenotypic reaction of the *Malus* genotypes investigated (Rohr et al. 2020).

Kanfra et al. (2018) grew the apple rootstock M26 in a sterilized matrix of perlite and sand, which was inoculated with a suspension of nematodes isolated from replant–diseased soil. The roots developed a typical ARD morphology and the concentrations of noraucuparin (**2**), noreriobofuran (**54**), 2-hydroxy-4-methoxydibenzofuran (**66**), and another tentatively identified dibenzofurans significantly increased (Kanfra et al. 2018).

As pointed out, replant-diseased soil stimulates apple roots to form biphenyls and dibenzofurans. The compounds are also exuded to the root surface and the surrounding soil. These findings support their role as phytoalexins in shaping the soil microbiome, which in turn influences the development and severity of ARD. However, the detailed effects of phytoalexins on the rhizosphere and soil microbial communities need to be investigated. This is also true for the possible cytotoxic effects of the phytoalexins on the producing apple roots. It also remains to be explored which specific ARD-related microbes induce the formation of the phytoalexins. The extent to which the phytoalexins inhibit the ARD causative microbes must likewise be investigated. The answers to all these questions will help understand the correlation between phytoalexin formation and ARD development. This knowledge may finally help establish measures for alleviating the ARD syndrome.

## Antimicrobial activity against phytopathogens and structure–activity relationships

Biphenyl and dibenzofuran phytoalexins differentially inhibit spore germination, germ tube development, and mycelial growth of various phytopathogenic fungi. Aucuparin (**3**) inhibited the spore germination rate of *Colletotrichum lindemuthianum* (black spot disease-causative agent) by 100% at 100 µg ml^−1^ (Watanabe et al. 1982). At the same concentration, it inhibited the spore germination rate of *Pestalotia funereal* (loquat gray leaf spot-causative agent) by 70% and reduced its germ tube length to 5 µm compared to the control (210 µm) after 24 h of inoculation. This activity was significantly reduced after 48 h, suggesting that aucuparin was inactivated by *P. funereal*, which may account for the pathogenic character of this fungus against loquat (Watanabe et al. 1982). Eriobofuran (**53**) was tested against eight plant pathogenic fungi and completely inhibited spore germination and germ tube growth of *P. funereal* at 43.2 µg ml^−1^ (Miyakodo et al. 1985). 2'-Methoxyaucuparin (**7**) and 4ʹ-methoxyaucuparin (**9**) reduced the spore germination rate of *Pestalotia photiniae* (leaf spot-causative agent) at 40 µg ml^−1^ by 64% and 71%, respectively (Widyastuti et al. 1992). Moreover, rhaphiolepsin (**8**) exhibited significant inhibitory activity against *Pestalotia* sp. because it was able to strongly reduce the spore germination rate and the germ tube length at 10 µg ml^−1^ (Watanabe et al. 1990; Widyastuti et al. 1991b). 4ʹ-Methoxyaucuparin (**9**) exhibited a significant effect on the hyphal growth of *Pestalotia* sp. at 40 µg ml^−1^ (Watanabe et al. 1990; Widyastuti et al. 1991a). The phytoalexins were more active against spore germination of *Pestalotia* sp. with increasing oxygenation (**3** vs **7**, **8**, **9**, and **53**). Furthermore, phytoalexins with a furan ring, as in eriobofuran (**53**), were more active than the biphenyls tested (**3, 7, 8,** and **9**), which may be related to the accessibility to the microbial target site.

The phytoalexins **3**,** 6**,** 7**,** 9**, **47–51**, **53**, **55**, **57**, and **61–63** affected the spore germination rate of *Alternaria alternata* (leaf spots, rots, and blight-causative agent), *Botrytis cinerea* (gray mold-causative agent), and *Fusarium culmorum* (seedling blight, foot rot, ear blight, stalk rot, and common root rot-causative agent) with ED_50_ values in the range of 12–328 µg ml^−1^. The dibenzofurans showed higher antifungal activity than the tested biphenyls, which was hypothesized to be related to the greater potential to penetrate the biological membrane and interfere with fungal growth (Kokubun et al. 1995a, b, c, d; Harborne 1997). Malusfuran (**56**), a dibenzofuran detected only in *M. domestica,* inhibited spore germination of* V. inaequalis* (scab-causative agent) at 1 mM. Interestingly, its aglycone, 2,4-dimethoxy-3,9-dihydroxydibenzofuran, showed higher toxicity to *V. inaequalis* than malusfuran. It inhibited spore germination of *V. inaequalis* at 0.5 mM, suggesting that the aglycone may be the actual phytoalexin (Hrazdina et al. 1997). Noraucuparin (**2**) and eriobofuran (**53**) had significant inhibitory activities against *Physalospora piricola* (fungal pathogen of apple and pear trees) with MIC values of 6.25 and 3.13 µg ml^−1^, respectively (Li et al. 2019).

Noraucuparin (**2**), aucuparin (**3**), and eriobofuran (**53**) inhibited conidial germination of *V. inaequalis* at 5 µM by 28%, 46%, and 34%, respectively. Interestingly, a combination of the three compounds synergistically inhibited the conidial germination of *V. inaequalis* by 74% (Sarkate et al. 2018). This report is the only study to explore the synergistic effects of biphenyl and dibenzofuran phytoalexins upon combination. Biphenyls **2**, **3**, **4**, **6**, **17**, **18**, **19**, and **20** inhibited the *Alternaria tenuissi* growth at 100 µg ml^−1^ by 48.5, 51, 54, 37, 50.3, 66.4, 58.8, and 33.3%, respectively (Song et al. 2021). In addition, 4ʹ-hydroxy-3,4,5-trimethoxybiphenyl (**18**) and 4ʹ,3,4,5-tetramethoxybiphenyl (**19**) showed inhibitory activities against four crop pathogens, i.e. *Fusarium graminearum* (stem rot-causative agent), *Helminthosporium maydis* (southern corn leaf blight-causative agent in maize), *Sclerotinia sclerotiorum* (vegetable sclerotinia-causative agent) and *Exserohilum turcicum* (northern corn leaf blight-causative agent in maize) at 100 µg ml^−1^, with *F. graminearum* being inhibited up to 63% by 4ʹ-hydroxy-3,4,5-trimethoxybiphenyl (**18)**. 4ʹ,3,4,5-Tetramethoxybiphenyl (**19)** affected the growth of *F. graminearum* and *E. turcicum* with inhibition rates of 59.4 and 58.8%, respectively (Song et al. 2021). This study preliminarily concluded that biphenyl substitution at C-4ʹ increased the antifungal activity of 4ʹ-hydroxy-3,4,5-trimethoxybiphenyl (**18**) and 4ʹ,3,4,5-tetramethoxybiphenyl (**19**) as compared to 2ʹ-hydroxy-3,4,5-trimethoxybiphenyl (**17**) and 2ʹ-hydroxyaucuparin (**6**). In addition, a methoxy substitution usually showed more potent antifungal effect than a hydroxy substitution in the same position, as exemplified by 3,4,5-trimethoxybiphenyl (**4**) and 3,4,5-trihydroxybiphenyl (**20**). In another study, the phytoalexins **14**, **15**, **64**, and **65** were tested against ten plant pathogenic fungi, sorbusin A (**64**) showing strong inhibitory activity against *Alternaria longipes* (brown spot-causative agent in tobacco) with a MIC value of 12.5 µg ml^−1^ (Gao et al. 2021).

It should be noted that only a single study reported the inhibitory activity of biphenyl and dibenzofuran phytoalexins on phytopathogenic bacteria. When a set of thirteen biphenyls and four dibenzofurans was tested against a panel of *E. amylovora* strains (fire blight disease-causing bacterium), noraucuparin (**2**), aucuparin (**3**), 3,5-dihydroxybiphenyl (**10**), 2,4-dihydroxydibenzofuran, eriobofuran (**53**), and noreriobofuran (**54**) exhibited antibacterial activity with MIC_50_ values equal to 43, 138, 17, 50, 195, 173 µg ml^−1^, respectively. Interestingly, a general tendency regarding structure–activity relationship was observed in this study. Biphenyls had somewhat stronger antibacterial activity than the structurally related dibenzofurans, e.g. 3,5-dihydroxybiphenyl vs. 2,4-dihydroxydibenzofuran, noraucuparin vs. 3,4-dihydroxy-2-methoxydibenzofuran, and aucuparin vs. eriobofuran (Chizzali et al. 2012a).

The above observations indicate that biphenyls and dibenzofurans possess antimicrobial activity against various plant pathogens. They appear to play a role in enhancing the resistance of Malinae species against pathogenic infections. However, all the above-mentioned antimicrobial studies lacked any information about the ecological concentrations of the phytoalexins. However, testing the compounds within their ecological concentrations is critical for understanding their role as part of the disease defense arsenal. Therefore, future works should consider the ecological concentrations of the phytoalexins to obtain reliable and informative data about their in vivo antimicrobial activity. Biphenyls and dibenzofurans need to be tested both individually and in combination against various phytopathogens to draw solid conclusions about their structure–activity relationships and possible synergistic effects. Studies of the antimicrobial activity of the phytoalexins may also be extended to human pathogens, thereby using the chance to possibly develop new human medicines. However, mechanistic studies are in demand to understand how the phytoalexins exert their antimicrobial activities.

## Conclusions

Biphenyls and dibenzofurans act as phytoalexins in the subtribe Malinae of the family Rosaceae upon induction by biotic and abiotic stimuli. They are preferably accumulated in the sapwood and their chemical structures are diverse. In a phylogenetic tree of the Malinae, their distribution is clade-related, and their substitution pattern is helpful for chemotaxonomy. However, the number of Malinae species explored for phytoalexins formation is still relatively small and it needs to be expanded.

Biphenyl and dibenzofuran phytoalexins exhibit differential antimicrobial activity against plant pathogenic fungi and bacteria. However, their antimicrobial activities have not yet been correlated with their ecological concentrations in specific plant tissues, which is of great interest to support their role as an efficient component of the intricate disease resistance arsenal. The insights into the synergistic effects of biphenyls and dibenzofurans against phytopathogens, their mechanisms of action, and their structure−activity relationships are still limited. Thus, many more biphenyls and dibenzofurans need to be diligently tested for their antimicrobial properties under ecological conditions before reliable conclusions can be drawn.

Although crucial biosynthetic steps leading to the formation of biphenyls were disclosed, dibenzofuran biosynthesis is largely unclear. More efforts should be undertaken to completely elucidate the metabolism of both biphenyls and dibenzofurans. This knowledge will finally provide a solid basis for manipulating phytoalexin formation using molecular breeding strategies, thereby increasing the disease resistance potential of economically valuable fruit trees, such as apple.

### *Author contribution statement*

BB and BL performed the literature search and drafted the manuscript. BL and LB critically revised the manuscript. All authors read and approved the manuscript.

## Data Availability

Data sharing is not applicable to this article as no datasets were generated or analyzed in the current study.
